# Fetal life malnutrition was not reflected in the relative abundances of adiponectin and leptin mRNAs in adipose tissue in male mink kits at 9.5 weeks of age

**DOI:** 10.1186/s13028-016-0250-3

**Published:** 2016-10-20

**Authors:** Connie F. Matthiesen, Anne-Helene Tauson

**Affiliations:** 1 Department of Veterinary Clinical and Animal Sciences, Faculty of Health and Medical Sciences, University of Copenhagen, Grønnegardsvej 3, 1870 Frederiksberg C, Denmark; 2 Department of Animal Nutrition and Management, Swedish University of Agricultural Sciences, Ulls väg 26, Uppsala, Sweden

**Keywords:** Fetal life malnutrition, Adipose tissue, Gene expression, Carnivores

## Abstract

**Background:**

Malnutrition in fetal life and during suckling have in some animal studies resulted in adaptive changes related to the fat and glucose metabolism, which in the long term might predispose the offspring for metabolic disorders such as obesity later in life. The objective was to study the effect of fetal life malnutrition in male mink on the gene expression of leptin and adiponectin in different adipose tissue sites.

**Results:**

Thirty-two male mink, strict carnivore species, exposed to low (FL) or adequate (FA) protein provision the last 16.3 ± 1.8 days of fetal life and randomly assigned to a low (LP) or adequate (AP) protein diet from 7 to 9.5 weeks of age were used. Adipose tissues (subcutaneous, perirenal and mesenteric) were analyzed using qPCR. Fetal life or post-weaning protein provision did not affect the relative abundances of leptin and adiponectin mRNAs in adipose tissue at 9.5 weeks of age. Relative abundances of leptin and adiponectin mRNAs were different between adipose tissue sites and were significantly higher in subcutaneous than in perirenal and mesenteric tissues.

**Conclusion:**

Fetal life protein malnutrition in male mink, did not result in adaptive changes in the gene expression of leptin and adiponectin mRNAs in adipose tissue at 9.5 weeks of age as found in rodents. However, both leptin and adiponectin mRNAs were significantly differently expressed between tissue sites.

## Findings

Malnutrition in utero can result in adaptive changes in the glucose and fat metabolism and may lead to increased fat deposition later in life. Exposing mink, a strict carnivore, to low protein provision in utero has resulted in lower birth weights [1, 2] and changes in the gene expression of fructose-1,6-biphosphatase, pyruvate kinase, fatty acid synthase and carnitine palmitoyl transferase, enzymes important for glucose [1, 3, 4] and fat homeostasis [5]. Present study investigated if the relative abundances of leptin and adiponectin mRNAs, both playing a key role in lipid and energy metabolism, were affected post-weaning by fetal life malnourishment.

Thirty-two male mink kits exposed to low (FL—14 % of metabolizable energy from protein—14P) or adequate (FA-29P) protein provision the last 16.3 ± 1.8 days of fetal life were used. The FL kits had significantly lower birth weight (10.3 g vs. 11.3 g; *P* = 0.004 [1]) than FA kits. An adequate protein provision was given from birth to weaning. At weaning, FL and FA males were randomly assigned to a low (LP-18P) or adequate (AP-32P) protein diet from 7 to 9.5 weeks of age giving four treatment groups (i.e. FA-AP, FA-LP, FL-AP, FL-LP). Dietary details are provided elsewhere [1, 4].

The experimental procedures followed the Danish National Legislation (license 2005/561-994), and was performed at the University of Copenhagen, Denmark.

At 9.5 weeks of age the males were anaesthetized by an intramuscular injection of 10.0 mg/kg BW Ketaminol and 2.0 mg/kg BW Narcoxyl (InterVet, Skovlunde, Denmark) and euthanized by excision of the heart. Tissues were collected, flash frozen and stored at −80 °C until analyses. The relative abundance of mRNA was estimated by quantitative real-time PCR using SYBR Green-I-detection and LightCycler 480 real-time PCR system (Roche Diagnostics, Copenhagen, Denmark). The tissue preparation, RNA extraction and RT-PCR program is described elsewhere [4]. The RT-PCR primers are listed in Table 1, and 18S rRNA was used as reference gene.

**Table 1:** Gene specific RT-PCR primers

Gene	Genebank	Sequence (5′–3′)	Length (bp)
Adiponectin	AB115956	5′TGGGATTGGAGAGTCGGGT3′ 5′ACTGGTCGTAGGTGAAGAGCA3′	216
Leptin	AB041360	5′TAGCCACATCCCTTTGAAGCA3′ 5′ATTCACATCCCTCACCTCCTG3′	243
18S rRNA	M10098.1	5′CGAGCCGCCTGGATACC3′ 5′CCTCAGTTCCGAAAACCAACAA3′	76

**Fig. 1:**
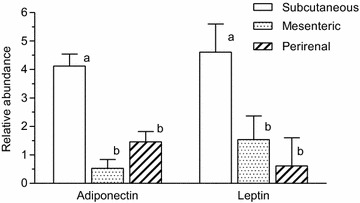
The relative abundances of leptin and adiponectin mRNAs. The relative abundances of adiponectin and leptin mRNAs normalized to 18 s rRNA in subcutaneous, mesenteric and perirenal adipose tissues from male mink kits exposed to fetal life low or adequate protein provision and fed a low or adequate protein diet post-weaning from 7 to 9.5 weeks of age

The relative abundance of leptin mRNA was not affected by FL protein provision which corresponded with the plasma leptin concentration and chemical body composition [4]. These results were supported by findings of the relative abundance of leptin mRNA in adult female mink [3] and the body composition of adult male mink [6] both protein malnourished in utero. This was in contrast to findings in male mice, protein malnourished in utero and during suckling, in which a lower body weight, circulating plasma leptin and abundance of leptin mRNA were found after weaning [7]. The relative abundance of adiponectin mRNA was not affected by fetal life or post-weaning protein provision similar to findings in rats exposed to protein restriction in fetal life and during suckling. However, if the rat offspring were fed a high energy diet post-weaning, the expression was significantly reduced [8]. The relative abundances of adiponectin and leptin mRNAs were both significantly different between adipose tissue sites (Fig. 1), having the highest expression in subcutaneous tissue. These differences were similar to findings in humans where the expression of leptin [9] and adiponectin mRNA were the highest in subcutaneous tissue [10, 11]. However, studies in lean rats have reported a higher abundance of adiponectin in visceral than in subcutaneous fat, whereas it was conversely in zucker diabetic rats [12].

In conclusion, fetal life protein malnourishment was not reflected in the abundance of leptin and adiponectin mRNAs similar to other findings in adult mink but in contrast to some findings in rodents, probably due to species differences and length of exposure to malnourishment. Both leptin and adiponectin mRNAs were differently expressed between tissue sites as found in other species.
